# The three syndromes and six Chinese patent medicine study during the recovery phase of COVID-19

**DOI:** 10.1186/s13020-021-00454-x

**Published:** 2021-06-07

**Authors:** Xuedong An, Liyun Duan, Yue Hong Zhang, De Jin, Shenghui Zhao, Rong Rong Zhou, Yingying Duan, Fengmei Lian, Xiaolin Tong

**Affiliations:** 1 Department of Endocrinology, Guang’anmen Hospital, China Academy of Chinese Medical Sciences, Beijing, 100053 China; 2 China Academy of Chinese Medical Sciences, Beijing, 100700 China; 3 Beijing University of Chinese Medicine, Beijing, 100029 China

**Keywords:** COVID-19, Recovery period, COVID-19 sequelae, Three syndromes and six Chinese patent medicines

## Abstract

The coronavirus disease 2019 (COVID-19), caused by severe acute respiratory syndrome coronavirus-2 (SARS-CoV-2), first broke out in Wuhan, China, in 2019. SARS-CoV-2 develops many types of mutations (such as B.1.1.7), making diagnosis and treatment challenging. Although we now have a preliminary understanding of COVID-19, including pathological changes, clinical manifestations, and treatment measures, we also face new difficulties. The biggest problem is that most COVID-19 patients might face sequelae (e.g., fatigue, sleep disturbance, pulmonary fibrosis) during the recovery phase. We aimed to test six Chinese patent medicines to treat three major abnormal symptoms in COVID-19 patients during the recovery phase, including cardiopulmonary function, sleep disturbance, and digestive function. We launched the “three syndromes and six Chinese patent medicines” randomized, double-blind, placebo-controlled, multicenter clinical trial on April 10, 2020. The results showed that Jinshuibao tablets and Shengmaiyin oral liquid significantly improved the cardiopulmonary function of recovering COVID-19 patients. Shumian capsules, but not Xiaoyao capsules, significantly improved patients’ sleep disorders. This might be because the indication of Xiaoyao capsules is liver qi stagnation rather than psychological or emotional problems. Xiangsha Liujun pills and Ludangshen oral liquid significantly improved digestive function. Our research provides a guideline for treating COVID-19 sequelae in patients during the recovery period based on high-quality evidence.

## Introduction

The coronavirus disease 2019 (COVID-19) caused by severe acute respiratory syndrome coronavirus-2 (SARS-CoV-2) first broke out in Wuhan, China, in 2019. The World Health Organization (WHO) declared the outbreak a public health emergency on January 30, 2020. As of January 15, 2021, there were more than 93 million confirmed infections, and the global death toll had reached 2,000,000, according to Johns Hopkins University [1]. Of these, severe/critically ill patients account for a large proportion of the deaths [2, 3]. Approximately 20% of COVID-19 patients developed pulmonary infiltration, some of which developed a severe form of the disease [4], especially the elderly and patients with underlying chronic diseases such as heart diseases and diabetes [5, 6]. Sepsis is the most common complication, followed by respiratory failure, acute respiratory distress syndrome (ARDS), heart failure, and septic shock [7]. As of February 11, 2020, the overall fatality rate of COVID-19 patients in China was 2.3%. However, the overall case fatality rate in patients aged 70–79 years was 8.0%, and it was 14.8% in patients aged ≥ 80 years [8]. We now have a preliminary understanding of COVID-19, including its pathology, clinical manifestations, and treatment.

## COVID-19

### SARS-CoV-2

SARS-CoV-2 is a positive-sense single-strand RNA virus of the Betacoronavirus lineage B and closely related to SARS-CoV [9]. There are no definitive conclusions as to the source of SARS-CoV-2. The genome sequence of coronaviruses carried by bats and pangolins was 90–96%, similar to SARS-CoV-2, but it is still uncertain whether any of these was the animal source of the first human infection [10].

### The pathology of COVID-19

COVID-19 transmission is primarily through the mouth by air, aerosol, contact, and feces [11]. Some studies have confirmed that SARS-CoV-2 could contaminate surfaces and medical equipment, especially in hospitals dedicated to COVID-19 patients [12]. After the human host is infected, SARS-CoV-2 infects alveolar epithelial cells by binding the angiotensin-converting enzyme II (ACE2) receptor. Binding to the receptor mediates the subsequent fusion between the virus envelope and the host cell membrane, allowing the virus to enter [13, 14]. By examining tissue samples from seven organs, including the lungs, spleen, liver, heart, kidneys, thyroid, and the testicles of patients who died from COVID-19 in 2019, it was found that the lungs of these patients had diffuse alveolar damage, pulmonary fibrosis, and neutrophil infiltration; fatty metaplasia, and sometimes infarction in the liver; myocardial edema and interstitial lymphocyte infiltration of the heart; and acute tubular injury in the kidneys [15]. These are related to a series of excessive reactions due to SARS-CoV-2 infection, including cytokine storm, immune system disorders, and abnormal blood coagulation. The expression levels of interleukin 1-B (IL-1B), interferon-γ (IFN-γ), chemokine-10 (IP-10), and monocyte chemoattractant protein 1 (MCP-1) are very high in COVID-19 patients [16]. Monocytes are circulating innate immune cells and might be the main participants in related pathologies in cytokine storm and COVID-19 [17]. Cytokine storm can cause ARDS or multiple organ failure outside the lungs and is an important factor leading to worsening of the disease or even death [18, 19]. Severe complications of COVID-19 include ARDS, embolism or pulmonary thrombosis, and a hypercoagulable state [20]. The incidence of alveolar capillary thrombosis in COVID-19 patients is nine times higher than in ordinary influenza patients [21]. Thrombocytopenia seems to be a key indicator of patient deterioration [22], and focal damage of pulmonary microvascular circulation is the primary mechanism of a fatal lung disease caused by SARS-CoV-2 [23]. Anticoagulation therapy was associated with improved prognosis in critically ill COVID-19 patients [24] who usually show enhanced coagulation and thrombosis [25, 26]. The mechanism might be the combined effect of the renin-angiotensin system and cytokine storm, which could mediate an increase in fibrinogen [27], extensive formation of microvascular thrombosis [28], disseminated intravascular coagulation (DIC), and secondary fibrinolysis [29].

### Clinical features of COVID-19

Common clinical manifestations of COVID-19 include fever (88.7%), cough (67.8%), fatigue (38.1%), sputum production (33.7%), shortness of breath (18.7%), sore throat (13.9%), and headache (13.6%) [30]. Moreover, when lung cells are infected with SARS-CoV-2, the effector CD4 + T cells reach the small intestine through the intestine-pulmonary axis, causing some gastrointestinal symptoms [11], including diarrhea (3.8%) and vomiting (5.0%) [30, 31]. Many cytokines enter through the blood–brain barrier and affect the central nervous system function, resulting in symptoms of the central nervous system [32]. However, all these symptoms do not seem specific, as some patients may develop a fever, while others may not [2, 33]. Critically ill patients might suddenly deteriorate in the later stages of the disease or during the recovery process. ARDS and multiple organ failure occur swiftly, leading to rapid death [34].

### Diagnosis, prevention, and treatment of COVID-19

Many technologies can be used to diagnose SARS-CoV-2 infection, including reverse transcription-polymerase chain reaction (RT-PCR), serological tests, point-of-care testing, smartphone monitoring of infectious diseases, nanotechnology-based methods, biological sensors, and metagenomic sequencing based on amplicons [35]. Saliva specimens could be considered a reliable and less resource-intensive alternative for screening asymptomatic SARS-CoV-2 infected patients [36].

COVID-19 prevention includes various forms of isolation and vaccination. The current treatment for COVID-19 is a bimodal therapy, one targets early infection and virus replication, and the other targets the systemic inflammatory phase of late infection and regulation [37], including host immune regulation, antiviral therapy, plasma therapy, anticoagulation therapy, ACE2 inhibitors, and steroid hormones. However, there is still no specific treatment, and "group immunity" cannot be achieved [22, 38–40]. Active symptom support is still the key for patients with mild to moderate disease, maintaining moisture and nutrition, and controlling fever and cough. Patients with severe infection or respiratory failure need to inhale oxygen through a face mask, high nasal flow, non-invasive ventilation, or mechanical ventilation. If all these prove ineffective, extracorporeal membrane oxygenation (ECMO) can be performed [41]. Currently, vaccination is the most promising preventive and treatment measure. In the 11 months since the SARS-CoV-2 and its genome were identified, the unremitting efforts of the scientific community have led to the development of more than 300 vaccine projects, and some vaccines have already been approved for emergency use. Available data indicate that new vaccine candidates might help protect individuals and reduce the spread of the pandemic [42].

### Emerging difficulties during the COVID-19 epidemic

#### Sequelae of COVID-19

At present, predicting the long-term effects of COVID-19, including the sequelae of the disease and the corresponding combined treatments, is still a challenge. The diverse presentations during the acute phase increase the difficulty in predicting the outcomes. Patients with severe diseases might suffer serious sequelae [43]. Studies have shown that more than three-quarters of COVID-19 patients report that they still have at least one symptom six months after the disease onset. The most commonly reported symptoms were fatigue or muscle weakness (63%), sleep disorders (26%), and anxiety or depression (23%). The patients’ lung function does not fully recover six months after the disease onset, and SARS-CoV-2 might infect again [44]. During severe inflammation, the wound healing response goes through a scarring process called fibrosis, leading to permanent tissue and organ damage. Fibrosis is a pathological wound healing response that leads to significant remodeling of the extracellular environment. It primarily leads to a decrease in tissue elasticity, resulting in dysfunction of organs, such as the lungs and heart, that depend on elastic characteristics to maintain normal function. Similar to SARS and Middle East Respiratory Syndrome (MERS), evidence indicates that SARS-CoV-2 infection could cause fibrotic damage, leading to complications after the viral infection period [45]. The use of corticosteroids in critically ill patients with COVID-19 appears to reduce the 28-day all-cause mortality and should be used when needed [46]. However, high-dose corticosteroid use might cause long-term distress in the recipients. One study followed 71 SARS survivors for 15 years. All were treated with short-term high-dose steroids at the severe stage of the disease. Of these, 15 cases of femoral head necrosis were considered to be related to steroid use, and four of them had mobility problems due to arthritis [47].

Chloroquine inhibits the production and release of tumor necrosis factor (TNF) and interleukin-6 (IL-6), suggesting that it could suppress the cytokine storm in COVID-19 patients [48]. However, current evidence failed to show the clinical benefits of prophylactic hydroxychloroquine over placebo or with no prevention, while the treatment shows a higher incidence of adverse events [49]. Hydroxychloroquine could cause neurological side effects, such as depression, psychosis, insomnia, manic episodes, and an increased risk of suicide [50, 51]. Treatment with it might weaken the host's immune response to SARS-CoV-2, especially in older patients or patients with comorbidities [52]. A study in Italy showed that the number of emergency room visits between February and April 2020 had decreased by 55%, compared to 2019, due to COVID-19. This indirectly led to an increase in patients requiring intensive care. The incidences of atrial fibrillation, ischemic heart disease, and severe renal insufficiency were higher in 2020 [53].

#### Mutation of SARS-CoV-2

Many variants of SARS-CoV-2 have appeared. These mutations usually do not have a decisive effect on the biological behavior of the virus and do not make a significant change in its structure and composition [54]. Variant B.1.1.7 of SARS-CoV-2 appeared in the United Kingdom in September 2020 [55]. Kyodo News reported on January 31, 2021, that the three SARS-CoV-2 mutations found in the United Kingdom (B.1.1.7), South Africa (B.1.351), and Brazil (P.1) have spread to at least 77 countries and regions around the world. The spike (S) protein of B.1.1.7 underwent a series of mutations, of which N501Y is one of the most important. The spike with this mutation binds more tightly to its cell receptor, ACE2 [56]. Current data indicate that B.1.1.7 does not escape the protection mediated by the Pfizer-BioNTech BNT162b2 COVID-19 vaccine [57]. After vaccination with the Moderna mRNA-1273 COVID-19 vaccine, neutralization against the intact B.1.351 variant was reduced but was still significant [58]. Japan’s National Institute of Infectious Diseases revealed that the existing vaccine is effective against B.1.1.7, but its effectiveness against the South African and Brazilian mutations is still unknown. Vaccine manufacturers suggest that they should improve the vaccines for the sake of prudence. The British pharmaceutical giant AstraZeneca stated that it has begun to improve its vaccine against the South African variant [59].

## The ‘three syndromes and six Chinese patent medicines’ project

Based on previous experience during the SARS and MERS epidemics and the current understanding of COVID-19, we focused on the prevalent COVID-19 sequelae because there are currently more than 90 million infected patients, and most might be affected by these. Consequently, we initiated the “three syndromes and six Chinese patent medicines” study on sequelae during the recovery phase of COVID-19.

### The implementation process of the ‘three syndromes and six Chinese patent medicines’ project

**Fig. 1:**
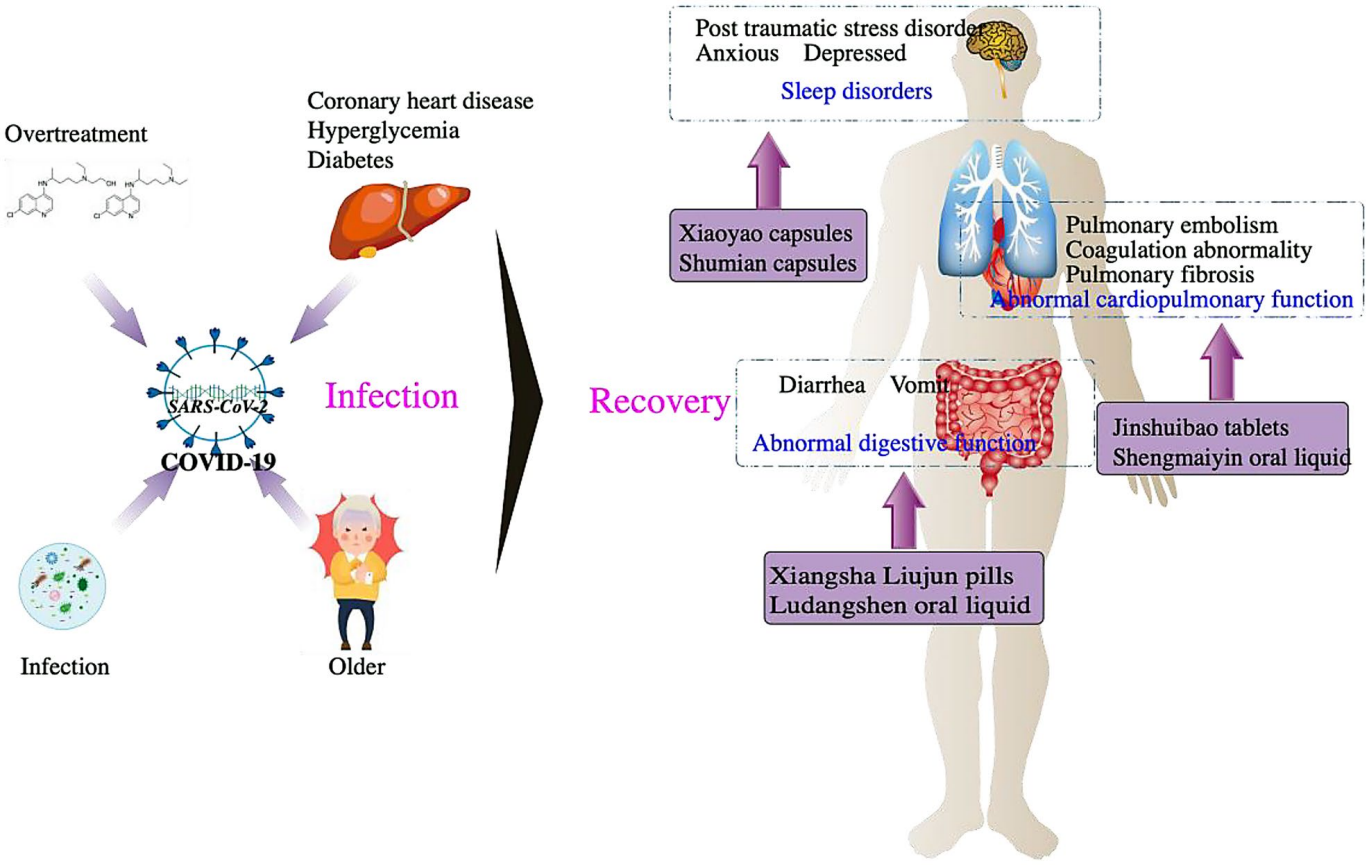
Six Chinese patent medicines played an important role in the three main symptoms during the recovery period of the COVID-19. During the COVID-19 period, due to virus infection, age, the existence of chronic diseases and over treatment, many abnormal symptoms and signs still existed in patients during the recovery period, including cardiopulmonary dysfunction such as pulmonary fibrosis, insomnia caused by anxiety and depression, vomiting, diarrhea and other digestive symptoms. In this regard, we have carried out the “three syndromes and six Chinese patent medicines” project, including Jinshuibao tablet, Shengmaiyin oral liquid could effectively improve cardiopulmonary function, Xiaoyao capsule and Shumian capsule could effectively improve sleep disorders caused by depression and anxiety, Xiangshaliujun pill and ludangshen oral liquid could effectively improve gastrointestinal symptoms such as diarrhea and vomiting.

Academician Tong Xiaolin (XT) arrived in Wuhan on January 24, 2020. On January 29, XT proposed that the Wuchang District Government and Hubei Provincial Hospital of Traditional Chinese Medicine jointly carry out community prevention and control activity using Chinese medicine. It was suggested that full coverage of traditional Chinese medicine (TCM) would significantly reduce severe and critical illness and death. For the recovery period, the medical treatment team leader of the State Administration of Traditional Chinese Medicine and XT held a kick-off meeting for the “three syndromes and six Chinese patent medicines” project on April 10. At the kick-off meeting, XT proposed new use for established drugs. Six already marketed Chinese patent medicines were screened through a randomized, double-blind, placebo-controlled, multicenter clinical trial for treating three primary symptoms—cardiopulmonary function, sleep disturbance, and digestive function, during the recovery phase of COVID-19. The trials was completed on October 13, 2020 (Fig. 1; Table 1).

**Table 1:** The main feature and outlook of “three syndromes and six Chinese patent medicines” project

Three Syndromes	Six Chinese patent medicines (Compose, Treatment duration)	Research method (included cases)	Main outcome	Improved symptoms	Outlook
Cardiopulmonary function	Jinshuibao tablet 9 Chinese Caterpillar Fungus; 2 weeks)	Randomized, placebo-controlled trial (experimental group:100; placebo group: 100)	Improvement in TCM symptom scores	Shortness of breath; sweating; chest tightness; dry cough	“Three syndromes and six Chinese patent medicines” project only reported the efficacy and safety results during the treatment period, without long-term follow-up. And the long-term biochemical indicators, imaging examination and clinical symptoms of patients, which is also the key content of our follow-up study is needed.
	Shengmaiyin oral liquid (Ginseng, Ginseng Root, radix ophiopogonis, and fructus schisandrae chinensis*;* 2 weeks)	Randomized, double-blind, placebo-controlled, multicenter clinical trial (experimental group:100; placebo group: 100)	symptom VAS score	chest tightness	
Sleep disorders	Shumian capsule (semen ziziphi spinosae, radix bupleuri, radix paeoniae alba, flos albiziae, cortex albiziae, bombyx batryticatus, periostracum cicadae, and medulla junci*;* 2 weeks)	Randomized, double-blind, placebo-controlled clinical study (experimental group:100; placebo group: 100)	Improvement in TCM symptom scores	Anxiety; insomnia; depression;	
	Xiaoyao capsule (radix bupleuri, radix angelicae sinensis, radix paeoniae alba, rhizoma atractylodis macrocephalae, poria, radix glycyrrhizae preparata, and herba menthae*;* 2 weeks)	Randomized, double-blind, placebo-controlled clinical trial (experimental group:100; placebo group: 100)	Improvement in TCM symptom scores	None	
Gastrointestinal symptoms	Xiangshaliujun pill (radix aucklandiae, fructus amomi, radix codonopsis, rhizoma atractylodis macrocephalae, poria, radix glycyrrhizae preparata, pericarpium citri reticulatae, rhizoma pinelliae, rhizoma zingiberis recens*,* Chinese Date, Jujube.; 2weeks)	Randomized, double-blind, placebo-controlled clinical trial (experimental group:100; placebo group: 100)	Improvement in TCM symptom scores	Fatigue; poor appetite; abdominal distension; loose stools	
	Ludangshen oral liquid (radix codonopsis; 2 weeks)	Randomized, double-blind, placebo-controlled, multicenter clinical study (experimental group:100; placebo group: 100)	Improvement in TCM symptom scores	Fatigue; anorexia; loose stool; shortness of breath;	

### Research of the three syndromes and six Chinese patent medicines

#### Treatment of abnormal cardiopulmonary function with traditional Chinese medicine

Acute cardiovascular complications, such as acute coronary syndrome, myocarditis, arrhythmia, and heart failure, have been widely reported in COVID-19 patients [60]. In a meta-analysis, the prevalence of myocardial injury in COVID-19 patients was 5%-38% [61]. In an observational cohort of 100 patients who recovered from COVID-19, 78% had abnormal cardiovascular magnetic resonance imaging 71 days after diagnosis. This finding was unrelated to previous comorbidities and most patients possibly had pericardial myocarditis [62]. Many COVID-19 survivors also have pulmonary sequelae [63–65]. Similar to SARS and MERS, the most apparent acute complication described in the early stages of the COVID-19 pandemic was severe lung disease, which required a high mechanical ventilation rate [2]. Pulmonary embolism caused by SARS-CoV-2 aggravated the burden of COVID-19 respiratory system damage [27]. Lung expansion ability continued to be impaired in 52% of SARS patients two years after infection [66]. A prospective cohort study in Beijing followed 71 SARS-infected medical staff for 15 years, showing that their lung function continued to be slightly impaired [47]. A major retrospective study of 143 patients in Italy found that 87.4% of patients still had at least one symptom, such as dyspnea or fatigue, two months after recovering from acute SARS-CoV-2 infection [67].

##### The effect of Jinshuibao tablets on patients with abnormal cardiopulmonary function during the recovery from COVID-19

The prospective, randomized, placebo-controlled trial included patients who recovered from COVID-19 from Hubei Provincial Hospital of Traditional Chinese Medicine, Wuhan Seventh Hospital, and Wuhan First Hospital between April 27, 2020, and May 15, 2020. The inclusion criteria were: met the three symptoms of hospital cure for more than two weeks and had an impaired cardiopulmonary function, shortness of breath, sweating, dry cough, chest tightness, palpitation, and decreased pulmonary and heart function. The diagnostic criteria were based on the China 7th edition of the Diagnosis and Treatment of COVID-19 Guidelines. The Ethics Committee of Hubei Provincial Hospital of Traditional Chinese Medicine approved the research protocol (HBZY2020-C27-01). Two-hundred patients were enrolled, 100 in the experimental group and 100 in the placebo group. The intervention measures were Jinshuibao tablets (fermented cordyceps powder, 0.33 g × 63 tablets/box). The patients received one tablet three times a day for two weeks, and the clinical outcomes were observed at 0, 1, and 2 weeks. Improvement in TCM symptom scores was compared between the two groups. The results showed that Jinshuibao tablets were better at reducing the symptom rates than the placebo, including shortness of breath (21/98 [21.43%] vs. 8/96 [8.83%], *P* = 0.011), sweating (29/78 [37.18%] vs. 15/74 [20.27%], *P* = 0.022), chest tightness (31/88 [35.23%] vs. 14/91 [15.38%], *P* = 0.002), dry cough (30/51 [58.82%] vs. 9/49 [18.37%], *P* < 0.001), and improve lung and heart function abnormalities.

Jinshuibao tablets are made of fermented *Chinese* *Caterpillar* *Fungus* powder that primarily contains nucleosides, bases, amino acids, sterols, and polysaccharides. The polysaccharides mainly contain 11 monosaccharide components, such as mannose and glucosamine [68]. Clinical studies have shown that Jinshuibao tablets alone or when combined with tiotropium bromide inhalants could significantly improve lung dysfunction caused by obstructive pulmonary disease, including improving lung ventilation [69, 70], reducing airway obstruction to prevent bronchospasm [71], and improving the pulmonary fibrosis symptoms in animal models [72, 73]. The medication might improve the patient's immune function by regulating peripheral blood Th17/Treg [70], significantly reducing the host's TNF-α and TGF-β1 levels [74], and inhibiting the accumulation of inflammatory cells in the lungs [75].

##### Effect of treatment with Shengmaiyin oral liquid on patients with cardiopulmonary abnormalities during the recovery phase of COVID-19

The randomized, double-blind, placebo-controlled, multicenter clinical trial included 200 COVID-19 convalescent patients with cardiopulmonary symptoms from Wuhan First Hospital, Wuhan Seventh Hospital, and Xiaogan Traditional Chinese Medicine Hospital from April 27, 2020, to May 14, 2020. All COVID-19 recovering patients were diagnosed and classified following the China 7th edition of the COVID-19 Diagnosis and Treatment Guidelines. The research plan was reviewed by the Ethics Committee of Wuhan First Hospital. The criteria for recovery were at least 72 h without fever, chest computed tomography (CT) showing substantial improvement in both lungs, clinical relief of respiratory symptoms, and two negative SARS-CoV-2 RNA pharynx tests obtained at least 24 h apart. Inclusion criteria were: aged 18–70 years, 2–4 weeks after discharge, and diagnosis of cardiopulmonary insufficiency, with two to three of the following clinical symptoms: shortness of breath, hyperhidrosis, chest tightness, palpitations, dry cough, or symptoms with a visual analog scale (VAS) score > 4. All participants signed informed consent forms. The intervention measures were Shengmai oral liquid that contained ginseng, *Ophiopogon* *japonicus*, and *Schisandra* (Tongrentang (Beijing) Co., Ltd., Beijing, China; National Drug Approval No. Z11020372). The patients received 10 mL of the fomulation three times a day for 2 weeks. The placebo group received a comforting agent following the same regimen. The cure standard was a symptom VAS score of zero after treatment. If the symptom VAS score decreased by 30% or more after treatment, the intervention was considered effective. Of the 200 patients, 192 completed the clinical observations. The results showed that the 2-week treatment with Shengmai oral liquid improved the chest tightness symptoms more than the placebo (75.00% vs. 55.56%, *P* < 0.05). The study also showed that Shengmai oral liquid effectively improved lung and heart function and the quality of life of patients recovering from COVID-19.

Twenty one Compounds of Shengmaiyin include 14 saponins, 6 lignans [76]. It is often used to treat chronic obstructive pulmonary disease [77], diabetic myocardium disease [78], and coronary atherosclerotic heart disease [79]. It exerts its action primarily by improving mitochondrial lipid metabolism and inhibiting oxidative stress damage [80]. Research on ginseng has mainly focused on saponins, polysaccharides, and volatile components. Among them, ginsenoside Rg1, the main component of ginseng, was shown to regulate various cell signaling pathways, including the c-Jun N-terminal kinase (JNK) pathway [81, 82], thus protecting the heart from various cardiovascular diseases. For example, it inhibits endoplasmic reticulum stress and autophagy to improve cardiac dysfunction caused by adriamycin [83]. Ginsenoside Re negatively affects cardiac contractility and autonomy and can cause changes in cardiac electrophysiological characteristics, which might be the reason for its antiarrhythmic effects [84]. Ginsenoside Rg1 effectively alleviated lung injury caused by sepsis, primarily by upregulating SIRT1 to alleviate endoplasmic reticulum stress and inflammation [85]. The main components of *O.* *japonicus* include steroidal saponins, high isoflavones, and polysaccharides, with various pharmacological activities, including anti-inflammatory and immune regulatory [86]. Three new isoflavone compounds isolated from the roots of *O.* *japonicus* were shown to alleviate the release of inflammatory chemokines in allergic bronchial asthma [87]. *Schisandra* is usually used to treat chronic cough. Its ethanol extract has a significant antitussive effect. It contains schisandrin and deoxyschisandrin, and it significantly weakens lung neutrophils and total inflammatory cells and increases the infiltration of TNF-α and interleukin-8 (IL-8) in the lungs [88]. *Schisandra* can also change the antioxidant status of the heart and improve its function [89].

#### Treatment of sleep disorders with traditional Chinese medicine

Previous evidence showed that some SARS patients have long-term psychological or memory abnormalities [90]. For example, in a cohort study of 99 SARS survivors, 33.5% were diagnosed with mental illness 30 months after infection, even though only 6% had a history of mental illness before SARS infection [91]. A meta-analysis of patients infected with SARS or MERS showed that 39% of them had post-traumatic stress disorder (PTSD), and 30–33% suffered from anxiety and depression [92]. The COVID-19 outbreak has affected physical health [93], and has induced a global mental health crisis, including mental illness and suicide [94–96]. Acute neuropsychiatric symptoms have been reported in 40–88% of patients with severe acute COVID-19 [97]. In a UK surveillance study of 125 unique COVID-19 cases, 77 experienced cerebrovascular events, and 39 had altered mental status [91]. In another study on 1,172 COVID-19 respondents recruited from 125 cities in China, anxiety and insomnia occurred in 33.02% and 24.66%, respectively. During the COVID-19 outbreak, physical and psychological symptoms are common in the general population [98]. The severity of insomnia was related to symptoms of depression and anxiety [99]. Research conducted during the initial stage of COVID-19 had highlighted its impact on mental health, with many people showing anxiety and depression [100, 101], facing the risk of post-traumatic stress disorder [102].

##### Effect of Shumian capsules on patients with sleep disorders during the recovery phase of COVID-19

The randomized, double-blind, placebo-controlled clinical study included 200 COVID-19 patients with sleep and emotional disorders in Hubei Provincial Hospital of Traditional Chinese Medicine from April to May 2020. Recovery of the COVID-19 patients was determined following the 7th edition of COVID-19 Diagnosis and Treatment Guidelines. The Ethics Committee of Hubei Hospital of Traditional Chinese Medicine examined and approved this study (No. HBZY2020-C27-01). Inclusion criteria were: meeting the diagnostic criteria for COVID-19 rehabilitation, cured and discharge for more than two weeks, and age between 18 to 70. All participants had sleep disorders, such as irritability, anxiety, or poor sleep, as the main clinical manifestations, with two symptoms appearing simultaneously or a single symptom graded as VAS ≥ 4, and all signed informed consent forms. The intervention was Shumian 0.4-g capsules (approval number: Z20050543), given orally, three tablets/capsules twice a day for two weeks. The control group received an equal dose of a placebo. The results showed that the anxiety symptom score of the experimental group after one week of treatment was significantly lower than the control group (3.00 ± 2.15 vs. 4.00 ± 2.06, *P* < 0.05). After two weeks of treatment, the treatment effectivity rate for insomnia (67/96 [69.79%] vs. 45/86 [52.33%], *P* = 0.004), anxiety (53/82 [64.63%] vs. 35/79 [44.30%], *P* = 0.006), and depression (43/66 [65.15%] vs. 26/65 [40.00%], *P* = 0.002) was higher in the experimental group than in the control group. Studies have shown that Shumian capsules significantly improved sleep and mood disorders due to COVID-19 during the recovery phase.

Shumian capsules are composed of *SEMEN* *ZIZIPHI* *SPINOSAE*, *RADIX* *BUPLEURI*, *RADIX* *PAEONIAE* *ALBA*, *FLOS* *ALBIZIAE*, *CORTEX* *ALBIZIAE,* *BOMBYX* *BATRYTICATUS,* *PERIOSTRACUM* *CICADAE*, and *MEDULLA* *JUNCI*. Jujube seed water extract can reduce insomnia symptoms by regulating the levels of monoamine and amino acid neurotransmitters in the brain [103]. Jujube is an effective ingredient in jujube seeds, which can enhance the hypnotic effect of pentobarbital and increase the sleeping time [104]. The *Bupleurum* formula is widely used to treat major depression [105]; it can improve the behavior of depression by regulating the metabolic profile and intestinal flora [106]. The active ingredient of Radix Paeoniae Alba has obvious antidepressant-like effects, which are closely related to the increase in hippocampal serotonin/adrenaline and the expression of brain-derived neurotrophic factor [107]. *Bupleurum* and Radix Paeoniae Rubra are among the most popular herbal pairs prescribed in TCM to treat depression. In animal models, the antidepressant effects of Radix Paeoniae Rubra and Radix Paeoniae Rubra were significantly better than those of Radix Paeoniae Rubra or Radix Paeoniae Rubra, achieved by regulating the mitogen-activated protein kinase (MAPK) signaling pathway. This conduction pathway and arachidonic acid metabolism have a synergistic effect [108]. Pretreatment with *Bupleurum* and Radix Paeoniae Alba significantly increased the concentration of serotonin and epinephrine in the hippocampus and cortical tissue [109]. *A.* *julibrissin* is used as a sedative in traditional oriental medicine. The flavonol glycosides quercitrin and isoquercitrin isolated from *A.* *julibrissin* increased sleeping time for pentobarbital-induced mice in a dose-dependent manner [110]. The anti-anxiety-like effects of *A.* *julibrissin* water extract are mediated by changes in the serotonergic nervous system, especially 5-HT1A receptors [111]. Stiff silkworms contain various chemical components, particularly proteins, peptides, and amino acids [112]. Its water-extract and alcohol-precipitate can effectively reduce the autonomous activities of mice and have sedative and hypnotic effects [113]. Cicada slough contains 17 hydrolyzed amino acids and various trace elements [114, 115]. Extracts with cicada slough, ethanol, and water showed anticonvulsant effects on pentylenetetrazol-induced convulsions in mice. The direct inhibitory effect of the water extracts was significant [116]. Rushes include luteolin, eriochlor, and vanillic acid [117]. Phenanthrene is the main effective component of *Juncus* *serrata* anti-anxiety effect [118], while the sedative and hypnotic effects of rushes ethyl acetate extract are the most obvious [119].

##### Effect of Xiaoyao capsules on patients with sleep disturbance during the recovery period from COVID-19

This randomized, double-blind, placebo-controlled clinical trial included 200 patients with sleep and mood disorders during their recovery from COVID-19, as defined in the 7th edition of the COVID-19 Diagnosis and Treatment Guidelines. Patients were recruited from April 1, 2020, to June 1, 2020. The inclusion criteria were: patients meeting the diagnostic criteria for the recovery phase of COVID-19 that were discharged from hospital for at least two weeks, had 2–3 symptoms of sleep or mood disorders, or a single symptom with a VAS score > 4, age 18–70 years. All participants provided written informed consent. Patients in the treatment group orally received four Xiaoyao capsules (0.34 g/capsule, China National Medical Products Administration approval no.: Z20050543) twice daily for two weeks. Patients in the control group received orally four Xiaoyao simulant capsules (0.34 g; placebo) twice daily for two weeks. Two weeks of Xiaoyao capsule treatment did not improve the symptom score over the control for irritability **(**4.43 ± 2.21 vs. 4.37 ± 2.11, *P* = 0.842), anxiety (4.93 ± 1.83 vs. 4.56 ± 1.74, *P* = 0.147), and poor sleep (5.53 ± 1.71 vs. 5.04 ± 1.66, *P* = 0.044). This study shows that Xiaoyao capsules cannot significantly improve the clinical symptoms of sleep and mood disorders during recovery from COVID-19.

Xiaoyao capsules are composed of radix bupleuri, radix angelicae sinensis, radix paeoniae alba, rhizoma atractylodis macrocephalae, poria, radix glycyrrhizae preparata, and herba menthae. Xiaoyao helps to soothe the liver and strengthening the spleen, nourishing blood, and regulating menstruation. It is often used clinically to treat sleep disorders caused by liver qi stagnation. Sleep disorders in this study patients were caused primarily by anxiety and irritability. This could explain why the Xiaoyao capsules failed to improve sleep and mood disorders in COVID-19 patients during the recovery phase.

#### Treatment of abnormal digestive function with traditional Chinese medicine

After SARS-CoV-2 infects the lung cells, the effector CD4 + T cells reach the small intestine following the intestine-lung axis, causing some gastrointestinal symptoms [11], including diarrhea (3.8%) and vomiting (5.0%) [30, 31]. Spleen and stomach dysfunctions are present in many COVID-19 convalescent patients. Approximately 5% of patients have abnormal digestive functions, including diarrhea or vomiting [44].

##### Effect of Xiangsha Liujun pills on patients with abnormal digestive function during the recovery period from COVID-19

This randomized, double-blind, placebo-controlled clinical trial recruited 200 patients with reduced digestive system function, including fatigue, loss of appetite, abdominal distension, and loose stool. All patients were in Hubei Province and during the recovery phase of COVID-19 as defined in the 7th edition of the COVID-19 Diagnosis and Treatment Guidelines. The inclusion criteria were: meeting the criteria for the recovery phase of COVID-19, cured and discharged from hospital for at least 2 weeks, aged 18–70 years. The main clinical manifestations were decreased digestive function symptoms such as fatigue, poor appetite, abdominal distension, and loose stools. Included patients had simultaneously at least three of these symptoms or had a single symptom with a VAS > 4. All participants signed informed consent forms. Patients in the experimental group were orally administered with Xiangsha Liujun pills, 12 pills three times a day for two weeks, half an hour before each meal. Eight pills contained the equivalent of 3 g of the original medicine. The control group received a placebo as prescribed by the physician, taken orally, one packet three times a day for two weeks, half an hour before each meal. Xiangsha Liujun Wan (concentrated pill; Zhongjing Wanxi Pharmaceutical Co., Ltd.; batch number: 200203). The results showed that providing Xiangsha Liujun pills as treatment for two weeks of significantly improved the symptoms of fatigue (70/80 [87.50%] vs. 58/83 [69.88%], *P* = 0.006), poor appetite (72/75 (96.00%) vs. 62/80 (77.50%), *P* < 0.001), abdominal distension (81/90 [90.00%] vs. 52/88 [59.09%], *P* < 0.001), and loose stools (37/40 (92.50%) vs. 25/52 (48.08%), *P* < 0.001).

Xiangsha Liujun pills are composed of radix aucklandiae, fructus amomi, radix codonopsis, rhizoma atractylodis macrocephalae, poria, radix glycyrrhizae preparata, pericarpium citri reticulatae, rhizoma pinelliae, rhizoma zingiberis recens, Chinese Date, *Jujube*. The chemical components of woody plants are mainly terpenes, alkaloids, anthraquinones, and flavonoids [120]. Muxiang decoction can accelerate gastric emptying and enhance the release of motilin [121]. Animal experiments have shown that different doses of the Muxiang decoction can promote gastric emptying and intestinal propulsion [122]. Brain-simmered woody can prevent diarrhea by maintaining the osmotic pressure and volume inside and outside the cells and the normal irritability of muscle nerves, and reducing the gastrointestinal tract excitability [123]. *Amomum* *villosum* contains as many as 138 chemical components, including bornyl acetate and camphor [124, 125]. *A.* *villosum* has a certain therapeutic effect on rats with functional dyspepsia and can promote gastric emptying and the release of substance P and motilin in gastric antrum tissue [126, 127]. *Codonopsis* contains sterols, glycosides, alkaloids, nitrogen-containing components, volatile oil, and a variety of inorganic elements and amino acids necessary for the human body [128]. The inulin-type fructan CP-A isolated from *Codonopsis* *pilosula* can significantly improve gastric mucosal injury, high ulcer index, and gastric mucosal bleeding injury in rats with acute gastric ulcers induced by ethanol [129]. *C.* *pilosula* water extract enhances the small intestine propulsion rate and serum motilin level in mice [130], an excitatory gastrointestinal hormone [131, 132]. *C.* *pilosula* polysaccharide treatment in rats can increase their appetite and food intake, and stomach pepsin excretion and activity [133]. The active ingredients of *Atractylodes* *macrocephala* include lactones, glycosides, polysaccharides, and amino acids [134]. Studies have shown that Baizhu promotes the proliferation of the beneficial bacteria *Bifidobacterium* and *Lactobacillus* in the intestinal flora and improves the intestinal flora status [135]. Atractylodes I has a strong role in enhancing salivary amylase activity, promoting intestinal absorption, and regulating intestinal function [136]. *Atractylodes* *macrocephala* stimulates the myoelectricity of the smooth muscle of the small intestine by exciting the M receptors of the gastrointestinal tract, thereby promoting gastrointestinal movement [137]. The main chemical components of *Poria* are triterpenoids and polysaccharides [138]. *Poria* decoction can directly relax isolated rabbit intestinal muscles and reduce the intestinal muscle contraction amplitude; it can prevent experimental gastric ulcers in rats and inhibit gastric juice secretion [139]. The main active ingredients of licorice extract include glycyrrhizin and glycyrrhetinic acid [140]. The decoction of raw licorice could reduce the tension of the spontaneous contraction of the intestine and could be used as a potentially therapeutic substance to improve gastrointestinal dysfunction [141]. The ethyl acetate extract of dried tangerine peel decoction is the strongest part of the digestion-promoting activity [142], and tangeretin and hesperetin can significantly promote the excretion of gastric juice and pepsin, improve the activity of pepsin, and enhance digestive function [143]. *Pinellia* is composed primarily of alkaloids and sterols [144]. The decoction and alcohol precipitation of *Pinellia* can inhibit gastric juice secretion in rats, reduce the free and total acids in the gastric juice, inhibit pepsin activity, protect the gastric mucosa, and promote mucosal regeneration [145]. Ginger contains many active substances, such as ginger essential oils, polysaccharides, sterols, and curcumin [146]. Curcumin significantly improved the activity of digestive enzymes in the small intestine of mice and promoted digestion. Additionally, it had a protective effect against gastric mucosal stimulation and chemical damage [147]. For example, ginger powder can significantly improve gastric mucosal damage caused by aspirin and has a protective effect on the gastric mucosa [148]. Jujube contains triterpenes, saponins, alkaloids, flavonoids, and glycosides [149]. Jujube polysaccharides, including glucose and fructose, can effectively reduce intestinal peristalsis time and contact with toxic and other harmful substances [150] and effectively treat constipation [151].

##### Effect of Ludangshen oral liquid on patients with abnormal digestive function during the recovery phase of COVID-19

The randomized, double-blind, placebo-controlled, multicenter clinical study recruited 200 patients at the recovery phase of COVID-19 following the 7th edition of the COVID-19 Diagnosis and Treatment Guidelines. Patients were treated at the Hubei Provincial Hospital of Traditional Chinese Medicine, Ezhou Traditional Chinese Medicine Hospital, and Xiaogan Traditional Chinese Medicine Hospital and had an abnormal digestive function. The Ethics Committee of Hubei Hospital for Traditional Chinese Medicine approved the trial (HBZY2020-C01-01). The inclusion criteria were as follows: meeting the diagnostic criteria of the COVID-19 recovery phase; at least two or three of the following TCM symptoms: fatigue, anorexia, diarrhea, loose stools, shortness of breath, and other symptoms related to decreased digestive function, or a single symptom with VAS score > 4; discharged from hospital for two to four weeks; aged 18 to 70 years; provided written informed consent. Patients in the intervention group were treated with Ludangshen oral solution (Shanxi Zhenglai Pharmaceutical Co., Ltd., Shanxi, China, production batch No. Z20059002), 10 mL twice daily for two weeks. Patients in the control group were treated with a placebo of Ludangshen oral solution (Shanxi Zhenglai Pharmaceutical Co., Ltd.), 10 mL twice daily for two weeks. The Ludangshen oral and placebo solutions were indistinguishable in flavor, taste, and appearance, including packaging color. Patients with comorbidities were allowed to undergo corresponding treatments. After two weeks of treatment, the symptom scores of fatigue (1.28 ± 1.45 vs. 3.01 ± 1.97, *P* < 0.001), anorexia (0.53 ± 1.08 vs. 1.49 ± 1.76, *P* < 0.001), loose stool (0.51 ± 1.44 vs. 1.19 ± 2.04, *P* = 0.009), and shortness of breath (0.89 ± 1.33 vs. 1.68 ± 2.22, *P* = 0.003) in the Ludangshen group were significantly lower than in the placebo group. Ludangshen oral liquid could be used as an optional treatment of abnormal digestive function in COVID-19 patients during the recovery phase.

As mentioned earlier, *Codonopsis* can effectively protect the gastrointestinal tract. Pharmacokinetic experiments showed that Lu Codonopsis increases the appetite [152]. *Codonopsis* regulation of the gastrointestinal function might be related to improvement in gastrointestinal motility and pepsin activity. The water extract of *Codonopsis* was reported to enhance the intestinal propulsion rate and serum motilin levels in mice [130].

## Outlook

At present, there are a large number of COVID-19 patients, more than 93 million confirmed infections. No matter in the COVID-19 infection stage or recovery stage, many high-quality studies had shown that the effect of traditional Chinese medicine is significant, and now there is a complete evidence chain [153, 154] about the prevention, treatment and recovery period of traditional Chinese medicine for COVID-19 [153, 154], that is, the role of traditional Chinese medicine is self-evident, and there are a large number of patients in recovery period in many regions, so more patients need to participate in the use of traditional Chinese medicine.

Previous studies on SARS suggested that the post infection effect of the virus would last for a long time. Our study of “three syndromes and six Chinese patent medicines” project only reported the efficacy and safety results during the treatment period, without long-term follow-up. We would track the biochemical indicators, imaging examination and clinical symptoms of patients, which is also the key content of our follow-up study.

## Conclusion

Many patients are presently recovering from COVID-19. Studies have shown that over three-quarters of COVID-19 patients report at least one symptom six months after the disease onset [44]. Fibrotic injury caused by SARS-CoV-2 could also lead to other complications [45]. Medications used during COVID-19 treatment, such as high-dose corticosteroids, might have long-term consequences, such as femoral head necrosis, arthritis, and inconvenience [47]. XT took the lead in launching the “three syndromes and six Chinese patent medicines” project on April 10, 2020, that screened six existing listed drugs through randomized, double-blind, placebo-controlled, multicenter clinical trials to treat symptoms of cardiopulmonary function, sleep disorder, and digestive function during the COVID-19 recovery phase. The results showed that Jinshuibao tablets and Shengmaiyin oral liquid significantly improved the patients’ cardiopulmonary function. Shumian capsules significantly improved the patients’ sleep disorders. Xiaoyao capsules wre ineffective for this indication, possibly because the cause, in this case, was liver Qi stagnation, not psychological or emotional disorders. Xiangsha Liujun pills and Ludangshen oral liquid significantly improved the patients’ digestive function. Our research provides a selection of treatment measures for disease sequelae in patients during the COVID-19 recovery phase based on high-quality evidence.

## Data Availability

Not applicable.

## Abbreviations

COVID-19: Coronavirus disease 2019
SARS-CoV-2: Severe acute respiratory syndrome coronavirus-2
WHO: World Health Organization
ARDS: Acute respiratory distress syndrome
ACE2: Angiotensin-converting enzyme II
IL-1B: Interleukin 1-B
IFN-γ: Interferon-γ
IP-10: Chemokine-10
MCP-1: Monocyte chemoattractant protein 1
RT-PCR: Reverse transcription-polymerase chain reaction
ECMO: Extracorporeal membrane oxygenation
MERS: Middle east respiratory syndrome
TNF: Tumor necrosis factor
IL-6: Interleukin-6
XT: Tong Xiaolin
TCM: Traditional Chinese medicine
CT: Computed tomography
VAS: Visual analog scale
JNK: C-Jun N-terminal kinase
IL-8: Interleukin-8
PTSD: Post-traumatic stress disorder
MAPK: Mitogen-activated protein kinase
